# A post-market cluster randomized controlled trial of the effect of the TENA SmartCare Change Indicator™ on urinary continence care efficiency and skin health in older nursing home residents

**DOI:** 10.1186/s13063-022-07031-z

**Published:** 2023-02-03

**Authors:** Muyibat Omotunde, Fredrik Agholme, Arne Böhling, Nicole Huige, Hardy Schweigel, Daniela Hayder-Beichel, Robert Reidy, Adrian Wagg

**Affiliations:** 1 Division of Geriatric Medicine, Department of Medicine, University of Alberta, Edmonton, AB Canada; 2 Essity Hygiene & Health AB, 405 03 Gothenburg, Sweden; 3 Essity/BSN Medical GmbH, Schützenstraße 1-3, 22765 Hamburg, Germany; 4 Essity/BSN Medical GmbH, Schützenstraße 1-3, 22761 Hamburg, Germany; 5 Department of Health, Hochschule Neiderrhein-University of Applied Sciences, Reinarzstr 49, 47805 Krefeld, Germany; 6 Staburo GmbH, Aschauer Strasse 26a, 81549 Munich, Germany

**Keywords:** Caregivers, Care aide continence care, Residents, Older adults, Nursing homes (long-term care facilities), TENA SmartCare Change Indicator, Medical device, Urinary incontinence, Digital health technology

## Abstract

**Background:**

Urinary continence care for residents of nursing homes who are unable to communicate their toileting needs usually involves care aides manually checking continence products (pads) to determine the level of urine saturation prior to changing. The TENA SmartCare Change Indicator is a medical device which estimates urine saturation and notifies caregivers of the optimal time for pad changes. This study will seek to examine the effect of the TENA SmartCare Change Indicator on urinary continence care efficiency and skin health, in comparison to usual care.

**Methods:**

This cluster randomized controlled trial (NCT05247047) involving older nursing home residents with urinary incontinence unable to consistently indicate their toileting needs, and their care aides, will compare technology-based and usual continence care over a period of 8 weeks. Co-primary endpoints of superiority in continence care efficiency and non-inferiority in the maintenance of skin health will be assessed. Secondary outcomes will examine the resident quality of life, sleep quality, responsive behaviours, changes in pad use and leakage episodes outside the pad. Change in care aide work engagement, job satisfaction and rushed tasks will be assessed. Benefits and challenges with the use of the device for continence care will be identified from the perspectives of the care staff.

**Discussion:**

Urinary continence assessment and care in nursing homes is reported as suboptimal and threatening to dignity. Data on the utility and effect of technological solutions for improving urinary continence care are few and conflicting. If shown effective, this technological solution has the potential to improve the care for older residents and improve the working lives of caregiving staff who look after this most vulnerable section of the population.

**Trial registration:**

ClinicalTrials.gov NCT05247047. Registration date is Feb 18, 2022

**Supplementary Information:**

The online version contains supplementary material available at 10.1186/s13063-022-07031-z.

Numbers in square brackets refer to SPIRIT checklist item numbers. The order of the items has been modified to group similar items (see http://www.equator-network.org/reporting-guidelines/spirit-2013-statement-defining-standard-protocol-items-for-clinical-trials/).

## Administrative information

**Table:** 

Title {1}	A post-market Cluster Randomized Controlled trial of the effect of the TENA SmartCare Change Indicator™ on urinary continence care efficiency and skin health in older nursing home residents
Trial Registration {2a,2b}	NCT05247047
Protocol Version {3}	2.1 January 27, 2022 Human Research Ethics Approval: Pro00115739 (Canada), approval pending (Germany)
Funding {4}	Essity Hygiene and Health AB
Author Details {5a}	Essity Hygiene & Health AB: Fredrik Agholme, Arne Böhling, Nicole Huige, Hardy Schweigel Staburo GmbH: Robert Reidy Hochschule Neiderrrhein- University of Applied Sciences: Daniela Hayder-Beichel Department of Medicine, University of Alberta: Muyibat Omotunde, Adrian Wagg
Name and Contact Information for the Trial Sponsor {5b}	Essity Hygiene and Health AB, SE-405 30 Gothenburg, Sweden
Role of Sponsor {5c}	co-creation of trial design, funding the clinical investigation, training visits with staff, data management, data analysis. Jointly responsible for report writing, interpretation of data, publication, and dissemination of results

## Introduction

### Background and rationale {6a}

Urinary incontinence (UI) amongst older adults affects individuals, their caregivers and the healthcare system as a whole [1]. The prevalence of UI is at its highest in the most vulnerable sector of our society, older adults in nursing homes (NH) [2]. UI is distressing and embarrassing for the individual, challenges personal dignity, increases the need for care and is associated with adverse health outcomes such as skin irritation, incontinence-associated dermatitis, urinary tract infection, isolation, depression and a reduced quality of life [3–7]. It is important that interventions in managing urinary incontinence are appropriate to abilities and disabilities of these older adults [8, 9]. Management of UI for NH residents is centred upon behavioural and conservative interventions [10, 11]. Given the high prevalence of cognitive impairment and physical disabilities in NH, only a minority of residents can be managed with interventions such as prompted voiding [12]. The majority are therefore managed by check and change routines with limited occasions when assisted toileting is offered. Check and change practices usually involve physical checking or assessment of pad saturation ‘wetness’ in order to judge whether a change is needed. Amongst residents who are unable to communicate their needs due to cognitive impairment, manual checks may trigger agitation [13, 14]. Suboptimal care practices in NH with deficiencies in both assessment and management of UI are also of concern [15, 16]. The use of sensor technology devices for continence care may increase residents’ comfort, promote dignity, lead to an improved quality of life and facilitate workload reduction and better time management for care providers [17]. The potential for modern technology to improve continence assessment has been explored in few studies to date, with mixed results [18, 19]. This study aims to examine the use of sensor technology for the delivery of urinary continence care and skin health in older adults unable to reliably indicate their toileting needs.

### Objectives {7}

The primary aims of the study are to evaluate the clinical performance of the interventional device regarding the superiority of continence care efficiency (compound score consisting of wet checks, pad changes, leakages outside the pad with linen and clothes changes) and non-inferiority of skin health simultaneously.

## Methods: participants, interventions and outcomes

### Design {8}

The study is a multinational multi-site, prospective parallel open-label cluster randomized trial with comparator groups comprising control (usual care) and intervention (device). Clusters, at the level of the nursing home clinical care unit, will be allocated to either intervention or usual care in a 1:1 ratio.

### Study setting and nursing home eligibility {9}

The study will be carried out in nursing homes in Canada and Germany. Each nursing home will be registered with the appropriate administrative authority and have 90% of residents aged 65 or over. Nursing homes will be located within 100 km of each trial centre. The nursing homes will be either current users of TENA continence products or be willing to become users of TENA continence products for the purpose of the study.

### Inclusion and exclusion criteria for participating residents {10}

Residents must be:

- over 65 years of age
- have urinary incontinence (UI) managed with incontinence products with tapes, belt or pull-up-type products
- permanent (intended length of stay longer than 4 months) residents of the facility
- unable to consistently communicate their toileting needs
- unable to successfully toilet and change their pad without assistance
- be using or willing to use TENA containment products for the course of the study
- be part of a manual check and change plan of continence care
- be, if applicable, on a stable regimen of medications for UI
- be able to provide informed consent to participate or, if unable to do so, have a legal representative who is willing and able to provide informed consent on their behalf

### Exclusion criteria:

These include residents who:

- have frequent (daily) faecal incontinence (FI) in the pad or have severe problems with FI as determined by the study investigator
- have a life expectancy of fewer than 3 months, in the opinion of the resident’s physician of record, or be in receipt of palliative/terminal care
- have severe continence product-related skin problems, as defined by the Ghent Global Incontinence Associated Dermatitis (GLOBIAD) categorization 2B (skin loss and infection) [20]
- have an indwelling or external urinary catheter, or anuria
- are managed using another automated or digital health technology incontinence management device
- have responsive behaviours of sufficient severity, in the opinion of the care staff, to make participation impractical
- as judged by the investigator, have any other condition that makes participation in the clinical investigation inappropriate
- have participated in an investigational study of a drug, biologic or device within 30 days prior to entering the clinical investigation or planned during the clinical investigation
- are dependent on either alcohol or recreational drugs

### Inclusion criteria for participating NH caregivers:

Eligible caregivers will have:

- cared for the resident on at least 3 days during the week before scheduled data collection points
- worked in the nursing home for at least 3 months, to ensure familiarity with the resident

### Who will take informed consent? {26a}

Written informed consent will be obtained from the nursing home directors of care, residents or their legal surrogate decision-makers, and consent from caregivers by the researchers (research nurse, research assistant). For residents unable to give informed consent and whose surrogate has given consent, assent to participate will be gained from the resident by research staff (research nurse, research assistant).

### Explanation for the choice of comparators {6b}

UI management is suboptimal in many nursing homes. Check and change regimens are threatening to dignity and potentially intrusive. Usual care refers to the current practices prevalent in the care unit. For the most part, this comprises manual checking of pads by care staff. Often, this involves touching it to feel if it seems heavy or smells or looking at the pad to see if the wetness indicator on the pad has changed in colour. The introduction of this new technology, compared to usual care, will potentially lead to the more efficient delivery of care, resulting in improvements to care and resident quality of life.

### Intervention description {11a}

**Fig. 1:**
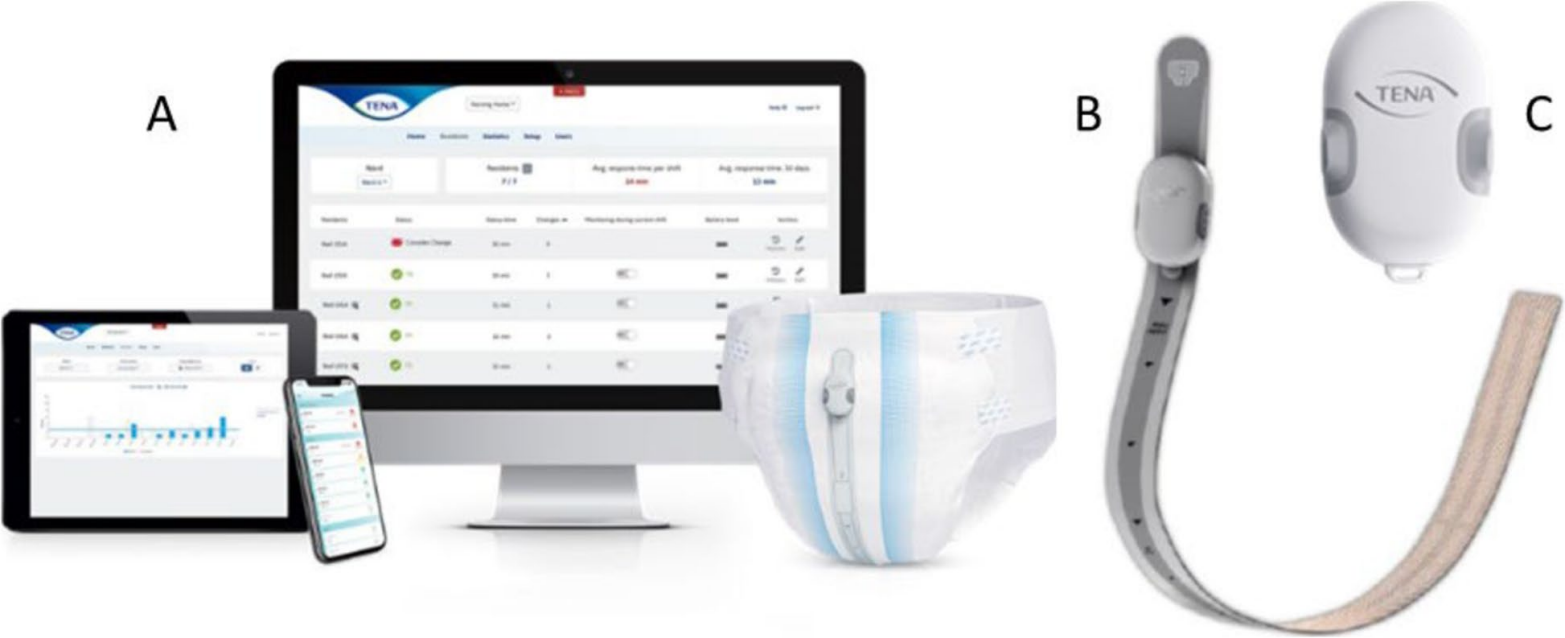
Components of the SmartCare Change Indicator: The electronic sensor (**B**) is placed on the outside of the incontinence product and estimates the urine saturation level. Results are displayed via an app and dashboard (**A**) installed on a mobile derive or desktop computer. **C** Detailed view on the transmitter which is combined with the sensor strip and placed on the incontinence product

The TENA SmartCare Change Indicator™ is a European Union/Canada class I medical device. The device is an accessory to absorbent continence products (pads) and is intended to be used on individuals with UI who are unable to consistently communicate their toileting needs. The device estimates the degree of urine saturation in the absorbent product and notifies a staff caregiver of the need to change the product. The device comprises a reusable electronic sensor and an application installed on one or more smart phones. The indicator monitors the saturation level of the absorbent continence product by using a sensor placed on the outside of the product (Fig. 1). The user interface shows the urine saturation level of the continence product. This information aids in decision support for caregivers to know when it is time to change the continence product, replacing a manual check routine. For the purposes of the trial, the level of saturation at which the sensor triggers is set at 70% and is not altered.

Prior to any resident procedures, data on the degree of person-centred continence care, the current check and change routine, and estimates for the cost of treatment of skin injuries and for prevention of skin health will be collected. On each intervention unit, a team from the sponsor will then conduct standardized training on the use of the interventional device . Researchers will familiarize care staff on trial**-**related procedures and data collection requirements.

### Criteria for discontinuing or modifying allocated interventions {11b}

Residents are free to discontinue participation in the clinical investigation at any time and are.

not required to give a reason for their decision. However, residents who discontinue the investigation will be asked about the reason(s) for their discontinuation and about the presence of any adverse event (AE)/adverse device effect (ADE) and, if possible, be assessed by an investigator.

Specific reasons for the withdrawal of *residents* are:

- the decision of a resident, or alternative decision-maker on behalf of the resident to withdraw from the investigation
- the investigator deems the resident unfit for the investigation

*Caregivers* (care aides, nurses) are free to discontinue participation in the clinical investigation at any time and are not required to give a reason for their decision. However, caregivers who discontinue the investigation will be asked about the reason(s) for their discontinuation.

Caregivers may be withdrawn from the clinical investigation and assessments at any time, if deemed necessary by the investigator.

*Care units* may be withdrawn from the study at the discretion of the investigator should he/she decide that the study procedures and protocols are not being adhered to and any action taken to remediate the situation results in no improvement.

### Strategies to improve adherence to interventions {11c}

Prior to involvement in the clinical investigation, investigators and relevant site staff will.

receive applicable investigational training. The training is conducted to ensure resident safety, compliance to protocol, adherence to the intervention applicable regulations/guidelines and accuracy of obtained clinical data. The investigators will ensure that appropriate training relevant to the clinical investigation is given to any other site personnel involved in the investigation and that new information of relevance to the performance of the investigation is forwarded to the staff involved.

### Relevant concomitant care permitted or prohibited during the trial {11d}

Usual and clinically indicated care is permitted during the study except for the initiation of a new medication for UI, which is prohibited for the duration of the trial. Any new treatments for skin health will be recorded. Any change in the clinical condition of participating residents during the trial will be recorded.

### Provisions for post-trial care {30}

No long-term risks of using the device are known or expected but these, if present, will be observed within this study. For all subjects who participate in this study, all AEs/ADEs and serious adverse events (SAEs) as well as other safety parameters will be followed up 14 days following trial cessation.

### Outcomes {12}

The co-primary and secondary outcomes for this study are shown in Table 1. The co-primary endpoints reflect the dual aims of use, preservation of skin health and efficient, optimized continence care delivery. The *care efficiency score* is the weighted sum of product checks, product changes, toilet visits, clothes changes and linen changes divided by the number of recorded diary days for each resident, reported as an average of the daily scores. The *skin health score* is defined as the average of the daily skin health grades. The validated Incontinence-Associated Dermatitis and its Severity Instrument has been adapted for use in this trial [21, 22].

**Table 1:** Endpoints

**Primary endpoint**	**Categories**	**Specifications**	**Baseline**
Care efficiency score	Clinical efficacy; observational level: resident; data type: continuous	Per resident, the average of the daily care efficiency scores. Explicitly, this is the weighted sum of the pre-defined care events (product checks, product changes, toilet visits, clothes changes, linen changes) divided by the number of recorded diary days of the resident in question. Only values from the final week of the intervention period will be considered The weights of the continence care tasks are provided in Table 3 depending on the group of patients and the time of day Typical endpoint values are expected to lie between 0 and 2000 Larger score values are to be interpreted as a greater workload for the staff caregiver in question and therefore less efficient	The baseline will be calculated in line with the main specification where only values from the final week of the baseline period (as outlined in 10.6) are to be considered
Daily skin health score	Clinical efficacy; observational level: resident; data type: continuous	Per resident, the average of the available skin health grades (1, 2, 3–4, 5) that have been reported daily in the study diary. Only values from the final week of the intervention period are to be considered The score values will be a real number between 1 and 5 Higher values are associated with a poorer skin health	The baseline will be calculated in line with the main specification where only values from the final week of the baseline period (as outlined in 10.6) are to be considered
**Secondary endpoints**	**Categories**	**Specifications**	**Baseline**
Daily skin health score	Clinical efficacy; observational level: resident; data type: continuous	Per resident, the average of the available skin health grades (1–2, 3–4, 5) that have been reported daily in the study diary. Only values from the final week of the intervention period are to be considered The score values will be a real number between 1 and 5 Higher values are associated with a poorer skin health	The baseline will be calculated in line with the main specification where only values from the final week of the baseline period (as outlined in 10.6) are to be considered
Product checks	Clinical efficacy; observational level: resident; data type: count	Sum of the product check events recorded in the study diary that occur during the final week of the intervention period for the resident in question	Sum of the product check events recorded in the study diary that occur during the final week of the baseline period for the resident in question
Product changes	Clinical efficacy; observational level: resident; data type: count	Sum of the product change events recorded in the study diary that occur during the final week of the intervention period for the resident in question	Sum of the product change events recorded in the study diary that occur during the final week of the baseline period for the resident in question
Toilet visits	Clinical efficacy; observational level: resident; data type: count	Sum of the toilet visit events recorded in the study diary that occur during the final week of the intervention period for the resident in question	Sum of the toilet visit events recorded in the study diary that occur during the final week of the baseline period for the resident in question
Clothing changes	Clinical efficacy; observational level: resident; data type: count	Sum of the clothing change events recorded in the study diary that occur during the final week of the intervention period for the resident in question	Sum of the clothing change events recorded in the study diary that occur during the final week of the baseline period for the resident in question
Linen changes	Clinical efficacy; observational level: resident; data type: count	Sum of the linen change events recorded in the study diary that occur during the final week of the intervention period for the resident in question	Sum of the linen change events recorded in the study diary that occur during the final week of the baseline period for the resident in question
Intermittent Skin Health Score Simple Skin Severity instrument [22]	Clinical efficacy; observational level: resident; data type: ordinal	The skin health grade recorded by the investigator at visit 4 Response an integer between 1 and 5 A larger skin health grade is associated to worse skin health	The skin health grade recorded by the investigator at visit 2
EQ-5D Index (25)	Clinical efficacy; observational level: resident; data type: ordinal	The EQ-5D index as recorded in visit 4 (individual resident’s study completion visit) Response is a natural number between 0 and 100 Higher values associated with a higher self-reported quality of life. E2-5D can be converted to utility scores for economic analysis	The EQ-5D index as recorded in visit 2 (individual resident’s end-of-baseline-period visit)
Quality of Life Score QoL-AD (26)	Clinical efficacy; observational level: resident; data type: ordinal	Quality of Life score recorded at visit 4. Response is between 13 and 52 Larger values are associated with a greater quality of life	Quality of Life score recorded at visit 2
Aggressive Behaviour Scale (27)	Clinical efficacy; observational level: resident; data type: ordinal	Given by the ‘Aggressive behaviour scale’ from the InterRAI Long Term Facility Assessment recorded in visit 4 Response is a real number between 0 and 12 Larger values are associated to greater aggression	Given by the ‘Aggressive behaviour scale’ from the InterRAI Long Term Facility Assessment recorded in visit 2
Sleep interruptions due to continence care	Clinical efficacy; observational level: resident; data type: count	Per resident, the total number of continence care episodes that are performed (as recorded in the diary) during the night (between 21:00 to 07:00 local time). Only values from the final week of the intervention period are to be considered	As given in the specification but considering values from the final week of the baseline period only
Total absorbency	Clinical efficacy; observational level: resident; data type: continuous	Per resident, the total number of products used multiplied by the known volume capacity of the product and summed. Only values from the final week of intervention are to be considered	As given in the specification but considering values from the final week of the baseline period only
UWES Total Score (28)	Clinical efficacy; observational level: cluster; data type: ordinal	UWES score as recorded in visit 4 Response is a natural number between 0 and 102 Larger values are associated with a greater work engagement	UWES score as recorded in visit 2
Organizational headroom	Clinical efficacy; observational level: cluster; data type: continuous	Per cluster, the assigned organization headroom score as recorded in visit 4 Response is a real number between 1 and 4	The assigned organization headroom as recorded in visit 2

### Participant timeline {13}

The following resident-level procedures will be conducted over 4 visits and a follow-up phone call. These are summarized in Table 2.

**Table 2:** Timeline and measures

Clinical investigation visit	Visit 1: resident inclusion	Visit 2: end of baseline	Visit 3: transition	Visit 4: completion and termination
Visit time point	Before start of baseline	Baseline 4 weeks (± 3 days) after start of baseline	Baseline + 2 weeks (± 3 days) after visit 2	Baseline + 6 weeks (± 3 days) after visit 2
Visit conducted by	Investigator/trial centre team	Investigator/trial centre team	Investigator/ trial centre team	Investigator/trial centre team
Nursing home eligibility	X			
Assessment of check and change procedures and person-centredness	X			
Assessments and procedures				
Investigational medical device				
Handover of TENA SmartCare Change Indicator and instructions to study site staff		X		
Confirmation of correct use of TENA SmartCare		X	X	X
Collection of investigational products				X
Adverse event/device deficiency documentation/follow-up		X	X	X
Resident				
Informed consent by resident or delegate	X			
Resident age, sex	X			
Resident medical and surgical history	X			
Resident concomitant medication review	X			X
Resident pad type use, with absorbent level, size and day/night use	X			
Resident functional impairment	X			X
Resident level of agitation and responsive behaviour	X	X		X
Resident quality of life and utility	X	X		X
Skin and skin care				
Assessment of skin health by an investigator	X	X	X	X
Assessment of skin health by a caregiver		Continuously in diary over complete study	Continuously in diary over complete study	Continuously in diary over complete study
Incontinence product				
Handover of single-use TENA incontinence products, as required	X			
Number of checks		Continuously in diary over complete study	Continuously in diary over complete study	Continuously in diary over complete study
Number of changes		Continuously in diary over complete study	Continuously in diary over complete study	Continuously in diary over complete study
Number of products used day and night		Continuously in diary over complete study	Continuously in diary over complete study	Continuously in diary over complete study
Absorption level of product used day and night		Continuously in diary over complete study	Continuously in diary over complete study	Continuously in diary over complete study
Timing of continence care episodes	X	X		
Leakage				
Outside product leakage episodes (soiled clothes and bedlinen)		Continuously in diary over complete study	Continuously in diary over complete study	Continuously in diary over complete study
Toileting behaviour				
Number of toileting episodes/24 h		Continuously in diary over complete study	Continuously in diary over complete study	Continuously in diary over complete study
Number of individually timed toileting episodes/24 h		Continuously in diary over complete study	Continuously in diary over complete study	Continuously in diary over complete study
Caregiver				
Informed consent by caregiver	X			
Work engagement	X	X		X
Organizational headroom	X	X		X

### Visit 1: baseline assessment

Evaluation of resident eligibility according to inclusion and exclusion criteria, obtaining of written informed consent from either resident or surrogate decision-maker, resident assent. Confirmation of resident eligibility and documentation of basic demographic characteristics (age, sex, comorbid conditions, coexistent medications).

Data on pad type, absorbance level, size and day or night use, skin health status, quality of life, responsive behaviours, care profile and functional assessment will be obtained (see measures below). Continence products, if needed, and resident-specific diaries will be provided to caregivers.

### Visit 2: end of baseline (4 weeks ± 3 days after start of baseline)

At this visit, a data quality check will be made and remedial action taken should this be needed. An assessment of the resident skin health status will be made, and confirmation of skin health eligibility will be made.

Written informed consent from care staff will be obtained and data on care staff work engagement, organizational headroom [3 questions on staff availability assessed on a 5-point Likert scale], tasks rushed and left undone on that shift and reported resident quality of life will be collected (see measures below). Investigational devices will be provided and installed.

### Visit 3: transition (2 weeks ± 3 days after visit 2)

Data quality check on documentation of performance parameters through the diary. An assessment of skin health status, documentation of safety, change in comorbid conditions and coexistent medications and confirmation of correct use of the device will be documented.

### Visit 4: completion (6 weeks ± 3 days after visit 2)

Data check on documentation of performance parameters through the diary, assessment of skin health status, reported resident quality of life, responsive behaviours as well as care profile and functional assessment, change in comorbid conditions and coexistent medications will be documented. Data on staff work engagement, organizational headroom and device safety parameters will be collected.

### Follow-up (8 weeks ± 3 days after visit 4)

A follow-up phone call to assess or follow up on any adverse events reported during the study.

### Qualitative assessment

A series of semi-structured interviews with staff having used the device will be undertaken to obtain feedback from the different stakeholders on the experience of working with the device, benefits seen/expected, any unintended consequences of use, suggestions for improvement and willingness to keep using it. All interviews are to be recorded, transcribed and then coded—independently by two researchers and then together where codes will be refined, combined and categorized to create themes and trends.

Sample size in each group is determined by saturation of themes—usually involving 5–12 interviews with different stakeholders.

Stakeholders are:

1. Nursing home manager/director, care manager, quality manager (management NH).
2. Nurse (registered nurse, licensed professional nurse).
3. Care aide (healthcare aide, personal support worker).

From the perspectives of these stakeholders, the benefit of the device is to be considered for the resident but also for the stakeholder.

### Sample size {14}

Due to the complexity of the study design with respect to power estimation, a sample size calculation was performed via simulation using SAS 9.4. Resident-level baseline and treatment profiles were generated according to best estimates of care event counts and skin health behaviours. Cluster-wide effects were added to the simulated responses such that an intra-cluster correlation (ICC) of 25% in both co-primary endpoints was present. Plausible values for the ICC were considered based on published results in Lee *et al.* [23].

A simulation set of 1000 hypothetical studies, with cluster sizes varying at random between 3 and 9 residents uniformly, was generated. Assuming an average decrease (i.e. improved efficiency) in the care efficiency score of 20% (σσ = 7.3%) as compared to usual care and an average reduction (i.e. improved skin health) of 0.2 (σσ = 0.3) in skin health grade as compared to usual care, a sample size of 16 clusters in total yields a power of 86.4%. To allow for cluster loss and to maintain a balanced study design, 20 clusters will be recruited, 10 in Canada and 10 in Germany. Based on the average site capacity, these 20 clusters correspond to approximately 180 individual residents. Caregivers will be recruited from the participating care units; we anticipate having at least 8 caregivers per cluster being able to contribute to the study or 160 caregivers in total.

### Recruitment {15}

NH will be recruited using a list of homes within 100 km of the trial centre in each country. The trial centre will prepare letters and information packages including guidance on the eligibility criteria and requirements for study participation. The director of care/site administrator or equivalent is the primary recipient of the initial information. One week after the first contact, the trial centre will follow up the contact and explain the study and address any questions. If the site is interested in participating, a visit will be arranged. Study team members will visit the site to provide further information about the study and answer questions. An agreement will be initiated and the site, subject to any required internal approval mechanism, will give written consent to be part of the study. Should further clarification or internal consultation be required, a follow-up telephone call not later than 1 week following the visit can be conducted. Following consent of the site and a written agreement, the site can be enrolled into the study by trial research staff. Care units will be randomly allocated from amongst the general clinical units in each home. Residents will be recruited following the identification of potential participants by unit staff. Caregivers will be recruited by the researchers from the participating care units.

### Assignment of interventions: allocation and sequence generation {16a}

Care units (clusters) at study sites will be randomly assigned 1:1 to the intervention and control arms. There will be one control or one intervention cluster at each study site. Randomization of NH and care units will be perfumed centrally using a computer-generated pseudorandom number table.

### Concealment mechanism {16b}

To mitigate contamination between intervention and usual care groups and in line with the trial design, each NH will only be allocated to a single intervention.

### Implementation {16c}

Randomization of NH and care units will be performed centrally by the sponsor using a computer-generated pseudorandom number table.

### Assignment of interventions: who will be blinded? {17a}

There will be no blinding at the level of the resident, clinical care unit or NH. The trial statistician will be blinded to unit allocation.

### Procedure for unblinding if needed {17b}

There is no blinding of the residents, clinical care unit or NH. The trial statistician will be unblinded after data analysis.

### Data collection and management

#### Plans for assessment and collection of outcomes {18a}

In addition to daily assessments of skin health and continence product usage, etc., data will be collected by researchers as outlined in the trial schedule (Tables 2, 3 and 4).

**Table 3:** Time estimate (in minutes) for each continence care task dependent on the resident persona (see Table 4). The table is divided into day and night (21:00–07:00 local time). Values are used as the weight when calculating the care efficiency score primary endpoint

Day	Group 1	Group 2	Group 3	Group 4	Group 5	Group 6
Pad check	0	0	1	6	6	6
Pad change	0	0	7	8	10	20
Change of clothes	0	5	8	10	18	40
Change of bedlinen	0	2	2	8	18	20
Toilet visit	0	0	10	18	20	50
Night	Group 1	Group 2	Group 3	Group 4	Group 5	Group 6
Pad check	0	0	1	4	4	4
Pad change	0	0	7	8	10	20
Change of clothes	0	5	8	10	18	40
Change of bedlinen	0	4	4	10	10	20
Toilet visit	0	0	10	20	0	0

**Table 4:**
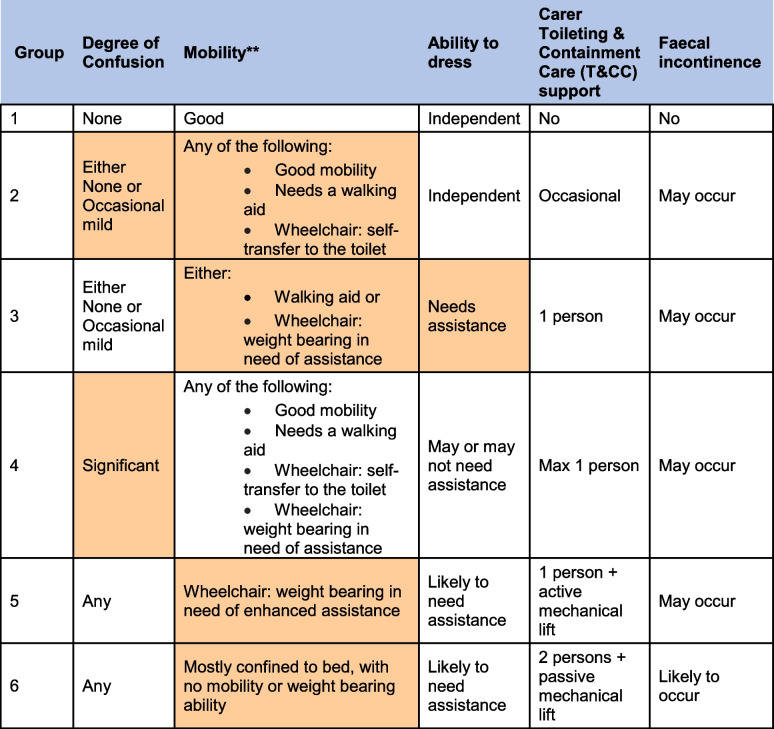
Personae and resident grouping depending on care need

Grouping of persons with functional urinary incontinence. Orange shading indicates the driver of care needs. In addition to functional incontinence caused by the inability to self-toilet due to cognitive and mobility issues, factors related to the bladder or bowel function may increase the need for toileting and containment care and influence a decision as to the need for containment products

**Includes standing, walking and transfers to and from the toilet, as well as the ability to carry out personal hygiene

#### Plans to promote participant retention and complete follow-up {18b}

All residents and caregivers will be given the opportunity to ask questions about the investigation and will be given sufficient time to decide whether to participate in the investigation or not. During the trial, no additional actions to promote resident retention will be taken other than caregiver staff support and researcher visits according to protocol. If any new important information arises during the clinical investigation, the resident will be informed both orally and in writing.

### Data management {19}

Resident data will be recorded directly into a custom-designed electronic case record file (eCRF). Data from paper records and diaries will be entered into the eCRF. Questionnaires will be considered source data. There will be no data entry into clinical records within the nursing home. Programming quality control processes will include checks for potential issues during import of raw data into the analysis software. A data validation plan (DVP) specifies the check that is to be performed on resident data for the clinical investigation. When all data from all endpoints of all residents have been entered, discrepancies solved and all reconciliation with the safety database is complete, the database will be locked, and the data will be analysed. Imputation methods will not be applied to the study data. No formal testing of outliers will be performed. All residents from all clusters will be pooled for analysis Data management and handling will be conducted according to the investigation-specific Data Management Plan (DMP) in accordance with applicable guidelines and standard operating procedures (SOPs). Any deviations, i.e. discrepancies and additions from the process defined in the DMP, will be described in an investigation-specific data management report (DMR).

### Confidentiality {27}

All data handling is in accordance with the European Union General Data Protection Regulation (GDPR) 2016/679 and the Canadian Personal Information Protection and Electronic Documents Act (PIPEDA). By fulfilling PIPEDA, the device also covers the Alberta Freedom of Information and Protection of Privacy Act and Health Information Act. All study data submitted to the sponsor will be de-identified. An identification list of participants will be kept separately and securely in password-protected, encrypted computer files.

### Statistical methods for primary and secondary outcomes {20a}

The primary analysis will be at the cluster level while some secondary endpoints are at the resident or caregiver level. The significance level for one-sided testing and confidence intervals is set to 2.5% while two-sided testing and confidence intervals are set to 5%.

Demonstration of the treatment effects on both primary endpoints is considered necessary for the demonstration of clinical benefit (co-primary endpoints).

The care efficiency and skin health co-primary endpoints are evaluated using linear mixed models (LMMs) to account for intra-cluster correlation using a random intercept effect. For each primary endpoint, the treatment effect is quantified using the difference in the least squares means of the treatment groups. For the care efficiency score, superiority is assessed by a one-sided test of the group mean difference where no superiority margin is added. For the skin health score, non-inferiority is assessed by a one-sided test of the group mean difference where a non-inferiority margin of + 0.5 will be used. Analysis will be per protocol. There are no plans for an intention-to-treat analysis.

### Methods for additional analyses (e.g. subgroup analyses) {20b}

Other endpoints will be assessed by superiority testing (intermittent skin health, daily skin health), LMM, controlled for country, baseline and clustering effects (QoL, EQ-5D, sleep interruption, responsive behaviours, total absorbency, UWES, organizational headroom, time spent in saturated pad, number of unnecessary checks).

Where the response is deemed to be insufficiently continuous to be assessed with a difference in averages, difference in frequencies will be tested instead. No formal interim analysis is planned.

### Method in analysis to handle protocol non-adherence and any statistical methods to handle missing data {20c}

All residents from all clusters are pooled for analysis which will be performed using the last observation carried forward where subjects are lost before the final observation. Data from subjects requesting data removal on their early termination will not be included in the analysis. Imputation methods will not be applied to the study data. No formal testing of outliers will be performed.

### Description of any interim analyses and stopping guidelines, including who will have access to these interim results and make the final decision to terminate the trial {21b}

There are no plans for an interim analysis and thus no stopping rules.

## Plans to give access to the full protocol, participant-level data and statistical code {31c}

The full protocol will be available on clinicaltrials.gov. Data and statistical code from this study shall be made available to researchers by application to the sponsor. There are no plans to make participant-level data or statistical code publicly available.

### Oversight and monitoring

#### Composition of the coordinating centre and trial steering committee {5d}

The trial steering committee comprises representatives from the sponsor, the chief investigator and site lead investigators. The trial steering committee will meet at least monthly to monitor trial progress and processes and act as necessary to ensure the trial timelines are adhered to. Trial centres, via the site lead investigator, have the responsibility for data collection and day-to-day study site management. The site leader and research staff will coordinate the study at the site and supervise the caregivers and residents with regard to all study requirements.

#### Composition of the data monitoring committee, its role and reporting structure {21a}

A Data Monitoring Committee (DMC) is not considered necessary for this post-market clinical investigation. The risk analysis indicates no need for a DMC. In case data retrieved during the clinical investigation contradict this decision, it might be reconsidered.

#### Adverse event (AE) reporting and harms {22}

All residents will be carefully monitored for the occurrence of AEs throughout the clinical investigation, from the first day to the completion of follow-up. The investigator or delegate will collect safety information using non-leading questions. Events directly observed or spontaneously volunteered by residents or caregiving staff will also be recorded throughout the clinical investigation. Clearly related signs, symptoms and abnormal diagnostic procedure results will be grouped together and reported as a single diagnosis or syndrome whenever possible. Any device-associated AE resulting from insufficient or inadequate instructions for use, deployment, implantation, installation, misuse, operation or malfunction will also be collected and recorded in the eCRF.

#### Frequency and plans for auditing trial conduct {23}

Every effort will be made to comply with the requirements of the trial protocol and the investigator is not allowed to deviate from the protocol. Waivers are prohibited. As required by national regulations or guidelines, requests for deviations and reports of deviations will be provided to the responsible Ethics Committee (EC) if the deviation affects resident’s rights, safety and well-being, or the scientific integrity of the clinical investigation. Under emergency circumstances, deviations from the protocol may proceed without prior approval by the sponsor and favourable opinion of the EC if the rights, safety and well-being of human residents need to be protected. Such deviations will be documented and reported to the sponsor and EC as soon as possible in accordance with national regulations. Regular trial monitoring visits will be performed by the clinical trial audit departments of the University Trial Centres.

Should a monitor or the sponsor identify non-compliance, this will be notified to the investigator in writing, with a request to correct the source of the deviation immediately. Corrective action will be implemented to avoid repeated non-compliance, which will usually include re-training and may include terminating the clinical investigation at the site. The sponsor is responsible for analysing deviations and assessing their significance. Corrective action(s) will be implemented to avoid repeated deviations, which may include suspending the clinical investigation at the investigation site or disqualification of the investigator. Major protocol deviations shall be reported immediately to the sponsor. Minor protocol deviations will be collected by the monitor during the monitoring visits. In case of any major protocol deviations, the designated monitors of the study will be informed by the investigator immediately upon detection.

#### Plans for seeking research ethics committee/institutional review board (REC/IRB) approval {24}

Ethics approval has been obtained from the University of Alberta Health Research Ethics Board (Pro00115739) and the ethics committee of the German Association for Nursing Science (22–034).

#### Plans for communicating important protocol amendments to relevant parties {25}

Reports of deviations will be provided to the responsible ethics committee if the deviation affects resident’s rights, safety and well-being or the scientific integrity of the clinical investigation. Participants will also be informed verbally and in writing of any amendments to protocols.

#### Additional consent provisions for collection and use of participant data and biological specimens in ancillary studies, if applicable {26b}

There are no additional consent provisions for use of data in ancillary studies. None is planned. No biological specimen will be collected as this study does not involve biological specimens.

#### Financial and other competing interests for principal investigation for the overall trial and each study site {28}

ASW: research support from Essity Hygiene & Health, AB, Pfizer Corp., speaker honoraria from Urovant Sciences.

FA, AB, HS and NH are employees of Essity Hygiene & Health.

RR is an employee of Staburo GmbH, contracted for this trial by Essity Hygiene & Health AB.

MO: no relevant competing interests.

#### Statement of who will have access to the final trial dataset, and disclosure of contractual agreements that limit such access for investigator {29}

The sponsor and investigators will have access to the final trial dataset. There are no contractual agreements that limit such access. Dissemination and use of the data will be jointly agreed by the sponsor and investigators.

The investigator will guarantee access to source documents for the trial monitor and auditors as well as for inspection by appropriate Regulatory Agencies/Competent Authorities, and Ethics committees (ECS), if required.

#### Dissemination plans {31a}

A final clinical investigation report (CIR) prepared by the sponsor will be completed, even if the investigation is prematurely terminated. All publications and presentations will be based upon the CIR. Publication of trial results will be jointly decided by the sponsor and trial investigators. Results will be disseminated via the traditional academic route and to the lay and professional press. Trial results will also be fed back to participating sites. Authorship and degree of involvement will be agreed with the investigators according to criteria published by the International Committee of Medical Journal Editors. While the sponsor has the right to use the results for registration, for internal presentation and for promotion, the investigators will be asked to comment upon and approve of any such use.

#### Authorship eligibility guidelines and any intended use of professional writers {31b}

AW is the chief investigator; he contributed to the design of the study and led the proposal and protocol development. FA, AB, MO and NH contributed to the study design and to the development of the proposal. RR was the lead trial statistician. All authors contributed to the writing and critical analysis and read and approved the final manuscript. There is no intended use of professional writers.

#### Model consent form and other related documentation given to participants and authorized surrogates {32}

Consent and assent forms for the study are available as supplementary data in Additional file 1.

#### Plans for collection, laboratory evaluation and storage of biological specimens for genetic or molecular analysis in the current trial and for future use in ancillary studies, if applicable {33}

Not applicable. There are no laboratory evaluations or use of biological specimens.

## Discussion

Care for UI can potentially be improved. A recent trial of a related device used to construct urinary continence care plans for residents was effective in reducing inappropriate pad use and improving resident quality of sleep, in addition to reducing the cost of care. Care aides opined that time was saved in care, allowing them to devote that time to other elements of care [REF ARCTICC]. The results of this trial have the potential to improve the care and quality of life for more dependent residents. Notwithstanding the effects of the COVID-19 pandemic, which has taken a great toll on older residents of nursing homes and the staff that look after them, there remains a great need to ensure that the care provided to this vulnerable group is of high quality. The vast majority of residents live with cognitive impairment, requiring multimodal person-centred support (social, medical, psychological) to maintain QoL [24]. Continence care, often neglected, forms an important part of this person-centred approach. Trials in NH are typically complicated, with multiple approvals required to implement research of this nature. The COVID-19 pandemic has made NH research difficult. All local protocols relevant to the pandemic will be followed to ensure safe trial conduct.

### Trial status

Protocol version 2.1 January 27, 2022.

Recruitment to commence in May 2022.

Trial completion is expected in March 2023.

## Supplementary Information

**Additional file 1.**

## Data Availability

Data and statistical code from this study shall be made available to researchers by application to the sponsor. The sponsor and investigators will have access to the final trial dataset. Dissemination and use of the data will be jointly agreed by the sponsor and investigators. Human research ethics approval was obtained from the Research Ethics Board, University of Alberta, Canada (Pro0011573), and the ethics committee of the German Association for Nursing Science (22–034). Informed consent from all participants or their legal surrogate decision-makers will be obtained.

## Abbreviations

ADE: Adverse device event
AE: Adverse event
CIR: Clinical investigation report
DMC: Data Monitoring Committee
DMP: Data Management Plan
DMR: Data Management Report
DVP: Data Validation Plan
EC: Ethics Committee
eCRF: Electronic case record form
FI: Faecal incontinence
GDPR: General Data Protection Regulation
GLOBIAD: Ghent Global Incontinence Associated Dermatitis scale
LMM: Linear mixed methods
NH: Nursing home
PIPEDA: Personal Information Protection and Electronic Documents Act
SAE: Severe adverse event
SOP: Standard operating procedure
UI: Urinary incontinence
