# Supplementary material for: A post-market cluster randomized controlled trial of the effect of the TENA SmartCare Change Indicator™ on urinary continence care efficiency and skin health in older nursing home residents
Source: Trials. 2023 Feb 3;24:80. doi: 10.1186/s13063-022-07031-z (PMC9895969; doi:10.1186/s13063-022-07031-z)
Supplement: Supplementary file 1 — Additional file 1. [file 13063_2022_7031_MOESM1_ESM.zip › assent form resident v2.0 04042022R2.docx]

**A postmarket cluster randomized controlled trial of the effect of the TENA SmartCare Change Indicator on continence care efficiency and skin health in long term care facilities**

WHEELS – ONE Pro 00115739

Principal Investigator(s): Dr. Adrian Wagg Phone Number: 780-492-5338

Study Coordinator: TBA Phone Number: TBA

**What is a research study?**

A research study is a way to find out new information about something. You do not need to be in a research study if you don’t want to. Your relative / person who makes decisions on your behalf has suggested that you might want to take part but we would like you to agree, if you can

**Why are you being asked to be part of this research study?**

You are being asked to take part in this research study because we are trying to learn more about how we can look after people with bladder problems better. We are asking you to be in the study because you have a bladder problem managed with diapers

If you join the study we will use a new type of diaper which lets your care staff know when it is time to change it. We hope that this will lead to you needing fewer changes and improve the care you get. Your care staff will also look at your skin daily to ensure that it isn’t being harmed by the new diaper.

We want to tell you about some things that will happen to you if you are in this study. You will have your skin looked at by a research staff member three extra times during the study. Your care staff will write down the number of pads you use and if they leak as well as other things about the care of your bladder. You will be asked to answer some questions about your health and how well you feel

You will be in the study for 10 weeks. You will have either the new diaper or your olde diapers for this time. This is so we can look at the effect of using the new diaper compared to the old one

**Will the study help you?**

We do not know whether the study will help you. We hope to find out. The results of the study will likely help you and others with bladder problems like yours

**What do you get for being in the study?**

You will not get anything for being in this study

**Do you have to be in the study?**

You do not have to be in the study. It’s up to you. No one will be upset if you don’t want to do this study. If you join the study, you can change your mind and stop being part of it at any time. All you have to do is tell us. It’s okay, the researchers and your relative / the person that helps you with your decisions won’t be upset.

**What choices do you have if you say no to this study?**

This study is extra, so if you don’t want to do it, nothing will change

**Who will see the information collected about you?**

The information collected about you during this study will be kept safely locked up. Nobody will know it except the people doing the research. The researchers will not tell your friends or anyone else.

**What if you have any questions?**

You can ask any questions that you may have about the study. If you have a question later that you didn’t think of now, either you can call or have your relative / the person that helps you with your decisions call us on 780-492-5338

**Other information about the study.**

If you decide to be in the study, please write your name below. You will be given a copy of this paper to keep.

Yes, I will be in this research study.

No, I don’t want to do this.

Name

Signature

Date

Person obtaining Assent

Signature

Date
