# Supplementary material for: A post-market cluster randomized controlled trial of the effect of the TENA SmartCare Change Indicator™ on urinary continence care efficiency and skin health in older nursing home residents
Source: Trials. 2023 Feb 3;24:80. doi: 10.1186/s13063-022-07031-z (PMC9895969; doi:10.1186/s13063-022-07031-z)
Supplement: Supplementary file 1 — Additional file 1. [file 13063_2022_7031_MOESM1_ESM.zip › WHEELS-ONE_ICF_caregiver 2.0 04012022R2.docx]

**WHEELS-ONE: Caregiver Informed Consent Form**

**Title:**  **A post-market cluster randomized controlled trial of the effect of the TENA SmartCare Change Indicator on continence care efficiency and skin health in long term care facilities.**

**Short title: Clinical investigation to evaluate TENA SmartCare Change Indicator in an institutionalized care setting**

# Consent for study participation

I have received written and verbal information about the purpose of the clinical investigation. I have read the written information and agree to take part in the study. I have had enough time to think about whether I want to take part in the study and have had the opportunity to ask all my questions about the clinical investigation which have been answered to my satisfaction. I am aware that participation is completely voluntary and that I can cancel participation at any time and without further explanation which will not influence my current or future work prospects or conditions.

| **Statement of agreement – study participation** | **Yes** | **No** |
| --- | --- | --- |
| I am aware that I am being asked to participate in a clinical investigation and that this includes following the instructions of my study team. | □ | □ |
| I have received a copy of, read and understood the corresponding information for caregivers. | □ | □ |
| The study has been comprehensively and understandably explained to me. In other words, I have, among other things, understood the risks and possibilities associated with study participation. | □ | □ |
| I have been given the opportunity to ask questions and all of these questions have been fully answered. | □ | □ |
| I am aware that participation in the study is voluntary and that I am free to discontinue the study at any time and that this will not affect my future work. | □ | □ |

Name of person to be contacted by the study staff in case I cannot be reached for follow-up.

| **Printed name** | **Contact details** |
| --- | --- |
|  |  |

After sufficient time for consideration, I voluntarily agree to participate in the above mentioned study by signing this form.

Study participant signature Place and date

Printed name

# Data Protection

In this Clinical Investigation, personal data, health information and medical findings are processed as sensitive personal data (hereinafter jointly referred to as “Data”. These Data are collected and stored in electronic and/or paper form by the local study team. Subsequently, your Data will be passed on to the study sponsor (Essity Hygiene and Health AB) in pseudonymized form. The Data will be transferd outside Canada to the EU. The purpose of collecting and passing this Data on is to allow it to be scientifically analysed to meet the objectives of the clinical investigation. No information identifying you will be transferred outside of the local study team. This Data will not be shared with your employer.

In order to verify the correct execution of the study it may be necessary for authorized representatives of the study sponsor, who are sworn to secrecy, as well as the competent supervisory authority to have access to your Data. Where appropriate, your Data may be stored for more than ten (10) years after completion or cancellation of the study.

To take part in the study, you are required to actively agree to the described collection, storage, use and retention of your data.

Particiaption in the study remains voluntary and may be discontinued at any time. Should the consent to participate in the study be revoked, you can decide whether the data stored up to this point will continue to be used for the study. If you do not, all information related to you will be deleted.

| **Statement of agreement – data protection** | **Yes** | **No** |
| --- | --- | --- |
| I have read and understood the information in the data protection section above. | □ | □ |
| I am aware that authorized persons sworn to secrecy, such as sponsor’s representatives, regulatory authorities and ethics committee representatives, may be granted access to my data, as well as the research notes documented in connection with the study available in my medical records, if this is required to verify the correct execution of the study. | □ | □ |
| I agree that my personal data is coded and managed in accordance with the EU General Data Protection Regulation (GDPR) 2016/679 and Canada´s Personal Information Protection and Electronic Documents Act (PIPEDA). Only the study doctor and involved study personnel will have access to the code key which makes it possible to identify me as an individual person. | □ | □ |
| I agree that the Sponsor may use the coded personal data and collected study data for the purpose of research, and that this information may be passed on to Sponsor’s affiliates, companies that collaborate with Sponsor and / or an authority (e.g. for scientific presentations or to improve the study device´s technical documentation or to support marketing purposes). I understand that this may mean that information collected as part of the study will be sent to other countries outside of Canada. If study data is sent outside of Canada, a sufficient level of protection must be ensured by referring to appropriate protective measures. | □ | □ |
| I am aware that data collected during the study may be processed by Sponsor or by companies contracted by Sponsor or its affiliates. Data collected may be processed and used even if I withdraw from the study, but I can ask for data to be corrected or deleted during the course of the clinical investigation. | □ | □ |
| I am aware that – should I no longer wish to participate in the study – I may revoke my consent to the processing of my pseudonymized data collected up to that point. | □ | □ |
| I am aware that my data will be scientifically analyzed and that the results from this study will be used in study reports, for scientific presentations and for publications. This under the condition that I will not be personally identifiable from my data. | □ | □ |
| I am aware that my data may be stored for more than ten years following the completion or cancellation of the study. | □ | □ |

By signing this form, I voluntarily consent to the processing of my sensitive personal data for the purpose described in the patient information.

Caregiver signature Place and date

Printed name

I certify that I have informed the caregiver about the purpose of the study, the applicable data protection regulations, and what it means to contribute. I further declare that to the best of my professional experience I have truthfully answered all questions concerning the above-mentioned study and that the caregiver has been properly and voluntarily consented. I will also make sure that the caregiver receives a copy of the signed consent form

Study doctor signature Place and date

Printed name
