# Supplementary material for: A post-market cluster randomized controlled trial of the effect of the TENA SmartCare Change Indicator™ on urinary continence care efficiency and skin health in older nursing home residents
Source: Trials. 2023 Feb 3;24:80. doi: 10.1186/s13063-022-07031-z (PMC9895969; doi:10.1186/s13063-022-07031-z)
Supplement: Supplementary file 1 — Additional file 1. [file 13063_2022_7031_MOESM1_ESM.zip › WHEELS-ONE_ICF_Resident v2 04012022R2.docx]

**WHEELS-ONE: Resident Informed Consent Form**

**Title:** **A post-market cluster randomized controlled trial of the effect of the TENA SmartCare Change Indicator on continence care efficiency and skin health in long term care facilities.**

**Short title: Clinical Investigation to evaluate TENA SmartCare Change Indicator in an institutionalized care setting**

# Consent for study participation

I have received written and verbal information about the purpose of the Clinical Investigation. I have read the written information, I understand the written information and I agree to take part in this Clinical Investigation. I have had enough time to think about whether I want to take part in the study and have had the opportunity to ask all my questions about the Clinical Investigation which have been answered satisfactory. I am aware that taking part is completely voluntary and that I can cancel my participation at any time and without further explanation. The above will not affect my future treatment and care.

| **Statement of agreement – study participation** | **Yes** | **No** |
| --- | --- | --- |
| I am aware that I am being asked to participate in a research study and that this includes following the instructions of my study doctor. | □ | □ |
| I have received a copy of, read and understood the corresponding resident information. | □ | □ |
| The study has been comprehensively and understandably explained to me. In other words, I have, among other things, understood the risks and possibilities associated with study participation. | □ | □ |
| I have been given the opportunity to ask questions and all of these questions have been fully answered. | □ | □ |
| I am aware that participation in the study is voluntary and that I am free to discontinue the study at any time and that this will not affect my future treatment and care. | □ | □ |

Name of person to be contacted by my study doctor in case I cannot be reached for followup.

| **Printed name** | **Contact details** |  |  |
| --- | --- | --- | --- |
|  |  |  |  |
|  | |  |  |
| I would like my primary care physician to be told about my participation in this study. | | Yes□ | No □ |

After sufficient time for consideration, I voluntarily agree and consent to participate in the above mentioned Clinical Investigation by signing this form.

Study participant signature Place and date

Printed name

# Data Protection

In this Clinical Investigation, personal data, health information and medical findings are processed as sensitive personal data (hereinafter jointly referred to as “Data”). These Data are collected and stored in electronic and/or paper form by the local study team. This Data is used for care categorization and evaluation of care efficiency. Further, the data will be used for study device assessment and to document care improvement as well as user and resident benefits. Subsequently, your Data will be passed on to the study sponsor (Essity Hygiene and Health AB, “Essity” or “Sponsor”) in pseudonymized form, which means that your personal identifiers have been removed and replaced with a unique code. The Data will therefore be sent out of Canada to the EU. No information identifying you will be transferred outside of the local study team. The purpose of collecting and passing this data on is to allow it to be scientifically analysed to meet the objectives of the clinical investigation.

In order to verify the correct execution of the study it may be necessary for authorized representatives of the study sponsor, who are sworn to secrecy, as well as the competent supervisory and regulatory authorities and research ethics boards to have access to your Data, in particular your health data. Where appropriate, your Data may be stored for more than ten (10) years after completion or cancellation of the study.

To be included in the study, you are required to actively agree to the described collection, storage, use and retention of your Data.

Participation in the study remains voluntary and may be discontinued by you at any time. Should your consent to participate in the study be revoked, you can decide whether Data stored up to this point will continue to be used for the study. If you do not agree that Data collected until your consent is revoked can be stored, your Data will be deleted.

| **Statement of agreement – data protection** | **Yes** | **No** |
| --- | --- | --- |
| I have read and understood the information in the data protection section above. | □ | □ |
| I am aware that authorized persons sworn to secrecy, such as Sponsor’s representatives, regulatory authorities and ethics committee representatives, may be granted access to my data, as well as the research notes documented in connection with the study available in my medical records, if this is required to verify the correct execution of the study. | □ | □ |
| I agree that my personal data is coded and managed in accordance with the EU General Data Protection Regulation (GDPR) 2016/679 and Canada´s Personal Information Protection and Electronic Documents Act (PIPEDA). Only the study doctor and involved study personnel will have access to the code key which makes it possible to identify me as an individual person. | □ | □ |
| I agree that the Sponsor may use the coded personal data and collected study data for the purpose of research, and that this information may be passed on to Sponsor’s affiliates, companies that collaborate with Sponsor and / or an authority (e.g. for scientific presentations or to improve the study device´s technical documentation or to support marketing purposes). I understand that this may mean that information collected as part of the study will be sent to other countries outside of Canada. If study data is sent outside of Canada, a sufficient level of protection must be ensured by referring to appropriate protective measures. | □ | □ |
| I am aware that Data collected during the study may be processed by Sponsor, its affiliates, or by companies contracted by Sponsor. Data collected may be processed and used even if I withdraw from the study, but I can ask for Data to be corrected or deleted during the course of the clinical investigation. | □ | □ |
| I am aware that – should I no longer wish to participate in the study – I may revoke my consent to the processing of my Data collected up to that point. | □ | □ |
| I am aware that my Data will be scientifically analyzed and that the results from this study will be used in study reports, for scientific presentations and for publications. This under the condition that I will not be personally identifiable from my Data. | □ | □ |
| I am aware that my Data may be stored for more than ten years following the completion or cancellation of the study. | □ | □ |

By signing this form, I voluntarily agree and consent to the processing of my sensitive personal data for the purpose described herein.

Study participant signature Place and date

Printed name

I certify that I have informed the study participant about the purpose of the study, the applicable data protection regulations, and what it means to participate. I further declare that to the best of my professional experience I have truthfully answered all questions concerning the above-mentioned study and that the study participant has been properly and voluntarily consented. I will also make sure that the study participant receives a copy of the signed consent form

Study doctor signature Place and date

Printed name
