# Supplementary material for: A post-market cluster randomized controlled trial of the effect of the TENA SmartCare Change Indicator™ on urinary continence care efficiency and skin health in older nursing home residents
Source: Trials. 2023 Feb 3;24:80. doi: 10.1186/s13063-022-07031-z (PMC9895969; doi:10.1186/s13063-022-07031-z)
Supplement: Supplementary file 1 — Additional file 1. [file 13063_2022_7031_MOESM1_ESM.zip › WHEELS-ONE_ICF_surrogate v2 04012022R2.docx]

**WHEELS-ONE: Designated representative Informed Consent Form**

**Title:** **A post-market cluster randomized controlled trial of the effect of the TENA SmartCare Change Indicator on continence care efficiency and skin health in long term care facilities.**

**Short title: Clinical investigation to evaluate TENA SmartCare Change Indicator in an institutionalized care setting.**

# Consent for study participation

I have received written and verbal information about the purpose of the clinical investigation. I have read the written information and agree to the participation of my family member / loved one. I have had enough time to think about whether I want my family member / loved one to take part in the study and have had the opportunity to ask all my questions about the clinical investigation which have been answered satisfactorily. I am aware that participation of my family member / loved one is completely voluntary and that I can cancel my family member / loved one’s participation at any time and without further explanation. The above will not affect their future treatment and care.

| **Statement of agreement – study participation** | **Yes** | **No** |
| --- | --- | --- |
| I am aware that my family member / loved one is being asked to participate in a clinical investigation and that this includes following the instructions of his/her study team. | □ | □ |
| I have received a copy of, read and understood the corresponding resident information. | □ | □ |
| The study has been comprehensively and understandably explained to me. In other words, I have, among other things, understood the risks and possibilities for my family member/ loved one associated with his/her study participation. | □ | □ |
| I have been given the opportunity to ask questions and all of these questions have been fully answered. | □ | □ |
| I am aware that participation in the study is voluntary for my family member / loved one and that I am free to discontinue the study at any time and that this will not affect their future treatment and care . | □ | □ |

Name of person to be contacted by my study doctor in case I cannot be reached for followup.

| **Printed name** | **Contact details** |
| --- | --- |
|  |  |

| I would like my family member / loved ones family (usual) physician to be told about his/her participation in this study. | Yes□ | No □ |
| --- | --- | --- |

After sufficient time for consideration, I consent to my family member / loved one’s participation in the above mentioned clinical investigation by signing this form. .

designated representative Place and date signature

Printed name

# Data Protection

In this Clinical Investigation, personal data, health information and medical findings are processed as sensitive personal data (hereinafter jointly referred to as “Data”). These Data are collected and stored in electronic and/or paper form by the local study team. This Data is used for care categorization and evaluation of care efficiency. Further, the data will be used for study device assessment and to document care improvement as well as user and resident benefits. Subsequently, your family member / loved ones Data will be passed on to the study sponsor (Essity Hygiene and Health AB, “Essity” or “Sponsor”) in pseudonymized form, which means that your family member / loved ones personal identifiers have been removed and replaced with a unique code. The Data will therefore be sent out of Canada to the EU. No information identifying your family member / loved one will be transferred outside of the local study team. The purpose of collecting and passing this data on is to allow it to be scientifically analysed to meet the objectives of the trial.

In order to verify the correct execution of the study it may be necessary for authorized representatives of the study sponsor, who are sworn to secrecy, as well as the competent supervisory and regulatory authorities and research ethics boards to have access to your family member / loved ones Data, in particular your family member / loved ones health data. Where appropriate, the Data may be stored for more than ten (10) years after completion or cancellation of the study.

To be included in the study, you are required to actively agree behalf of your family member / loved one to the described collection, storage, use and retention of their Data.

Participation in the study remains voluntary and may be discontinued on behalf of your family member / loved one by you at any time. Should your consent to participate in the study be revoked, you can decide whether Data stored up to this point will continue to be used for the study. If you do not agree that Data collected until your consent is revoked can be stored, your Data will be deleted.

| **Statement of agreement – data protection** | **Yes** | **No** |
| --- | --- | --- |
| I have read and understood the information in the data protection section above. | □ | □ |
| I am aware that authorized persons sworn to secrecy, such as sponsor’s representatives, regulatory authorities and ethics committee representatives, may be granted access to my family member / loved one’s data, as well as the research notes documented in connection with the study available in my medical records, if this is required to verify the correct execution of the study. | □ | □ |
| I agree that the personal data of my family member / loved one is coded and managed in accordance with the EU General Data Protection Regulation (GDPR) 2016/679 and Canada´s Personal Information Protection and  Electronic Documents Act (PIPEDA). Only the study doctor and involved study personnel will have access to the code key which makes it possible to identify my family member / loved one as an individual person. | □ | □ |
| I agree that the Sponsor may use the coded personal data of my family member / loved one and collected study data for the purpose of research, and that this information may be passed on to Sponsor’s affiliates, companies that collaborate with Sponsor and / or an authority (e.g. for scientific presentations or to improve the study device´s technical documentation or to support marketing purposes). I understand that this may mean that information collected as part of the study will be sent to other countries outside of Canada. If study data is sent outside of Canada, a sufficient level of protection must be ensured by referring to appropriate protective measures. | □ | □ |
| I am aware that data collected during the study may be processed by Sponsor or by companies contracted by Sponsor or its affilitates. Data collected may be processed and used even if my family member / loved one withdraw from the study, but I can ask for their data to be corrected or deleted during the course of the clinical investigation. | □ | □ |
| I am aware that – should my family member / loved one no longer wish to participate in the study – I may revoke their consent to the processing of Data collected up to that point. | □ | □ |
| I am aware that my family member / love one’s data will be scientifically analyzed and that the results from this study will be used in study reports, for scientific presentations and for publications. This under the condition that my family member / loved one will not be personally identifiable from my data. | □ | □ |
| I am aware that my family member / loved one’s data may be stored for more than ten years following the completion or cancellation of the study. | □ | □ |

By signing this form, I voluntarily consent to the processing of my family member / loved one’s sensitive personal data for the purpose described in the patient information.

designated representative Place and date signature

Printed name

I certify that I have informed the study participant, within her/his ability to understand, and her/his legally designated representative about the purpose of the study and what it means to participate. I further declare that to the best of my professional experience I have truthfully answered all questions concerning the above-mentioned study and that the legally designated representative has been properly and voluntarily consented. I also certify that the study participant and her/his legally designated representative receive a copy each of the signed consent form:

Study doctor signature Place and date

Printed name
